# Influence of carbapenem resistance on mortality of patients with *Pseudomonas aeruginosa* infection: a meta-analysis

**DOI:** 10.1038/srep11715

**Published:** 2015-06-25

**Authors:** Qianqian Liu, Xiaoqing Li, Wenzhang Li, Xinmiao Du, Jian-Qing He, Chuanmin Tao, Yulin Feng

**Affiliations:** 1 Department of Laboratory Medicine, West China Hospital of Sichuan University, Chengdu, Sichuan, 610041, China; 2 Collaborative Innovation Center of Sichuan for Elder Care and Health, Chengdu Medical College, Chengdu, Sichuan, 610500, China; 3 Department of Cardiology, First Affiliated Hospital of Chengdu Medical College, Chengdu, Sichuan, 610500, China; 4 Department of Respiratory Medicine, West China Hospital of Sichuan University, Chengdu, Sichuan, 610041, China

## Abstract

Treatment of infectious diseases caused by the carbapenem-resistant *Pseudomonas aeruginosa* (CRPA) is becoming more challenging with each passing year. We conducted a meta-analysis to assess the impact of carbapenem resistance on mortality of patients with *P. aeruginosa* infection. We searched PUBMED, Web of science, EMBASE, Google Scholar and the Cochrane Library up to December 25, 2014, to identify published cohort or case-control studies. 17 studies, including 6660 patients carrying *P. aeruginosa*, were identified. The pooling analysis indicated that patients infected with CRPA had significantly higher mortality than those infected with carbapenem-susceptible *P. aeruginosa* (CSPA) (crude OR = 1.64; 95%CI = 1.40, 1.93; adjusted OR = 2.38; 95%CI = 1.53, 3.69). The elevated risk of mortality in patients with CRPA infection was not lessened when stratified by study design, sites of infection, or type of carbapenem, except that the estimate effect vanished in CRPA high-incidence region, South America (crude OR = 1.12; 95%CI = 0.64, 1.99). Begg’s (z = 0.95, p = 0.34) and Egger’s test (t = 1.23, p = 0.24) showed no evidence of publication bias. Our results suggest that carbapenem resistance may increase the mortality of patients with *P. aeruginosa* infection, whether under univariate or multivariate analysis.

Being a glucose non-fermentative Gram-negative bacillus, with minimal requirements for survival and a considerable ability to adapt to various environmental challenges, *Pseudomonas aeruginosa* has become an increasingly notorious pathogen of nosocomial infection in recent years. And this pathogen is regarded as an unfortunate by-product of progress in the modern medical treatment^1^. Although there are successive advances in antimicrobial therapy, *P. aeruginosa* associated infection remains to be alarming, with mortality ranging from 18% to 61%^2^,^3^,^4^.

Owing to intrinsic resistance and rapid acquisition of additional resistance to commonly used antibiotics, the therapeutic options for *P. aeruginosa* infection are limited^5^. For *P. aeruginosa* infection, carbapenem may be only one of choice of antibiotic. As a result, the emerging of carbapenem resistance is of great concern. According to epidemiologic studies, the prevalence of carbapenem-resistant *P. aeruginosa* (CRPA) varied by geographic regions. Based on the data of the Carbapenem Antimicrobials *Pseudomonas* Isolate Testing At regional Locations (CAPITAL) surveillance program in 2010, the overall rates of CRPA isolates among all *P. aeruginosa* isolates ranged from 7.4% to 35.4%^6^. However, the proportion of carbapenem resistance in *P. aeruginosa* has reached even more than 60% in several other regions of the world, especially in South America^7^. As carbapenem resistance, partly through altering outer membrane proteins or via over-expression of efflux pumps, may enhance excretion of carbapenem and other agents, multi-drug resistance in CRPA is not surprising, which poses even more serious problem to the selection of anti-pseudomonal agents from limited arsenal^8^.

It is noted that the prevalence of CRPA has been increasing steadily in recent years, which exerts deleterious effects on clinical outcomes, such as length of hospital stay or health-care costs^9^,^10^. Complicated by the severity of the underlying disease and the virulence of the pathogens, the detrimental impact of carbapenem resistance on mortality of patients with *P. aeruginosa* infection, however, remains obscure^11^,^12^. Several researches showed higher mortality rates among patients infected with CRPA isolates^13^, while others didn’t repeated the same tendency^14^. In order to effectively guide antibiotic policy during empirical therapy and to prioritize future interventions to these types of drug-resistant infections, it is paramount to identify the effect of resistance on mortality. Although Nathwani D, *et al.* reported increased all-cause mortality among hospitalized patients with resistant and MDR P. aeruginosa infections in a recent meta-analysis, their reseach was limited by heterogeneity of definition of resistance and multi-drug resistance^15^. Up till now, there is no meta-analysis reported focusing on the clincal impact of CRPA associated infection. Considering this scenario, we performed a meta-analysis to determine the influence of carbapenem resistance on mortality of patients with *P. aeruginosa* infection.

## Results

### Flow of included studies

**Figure 1:**
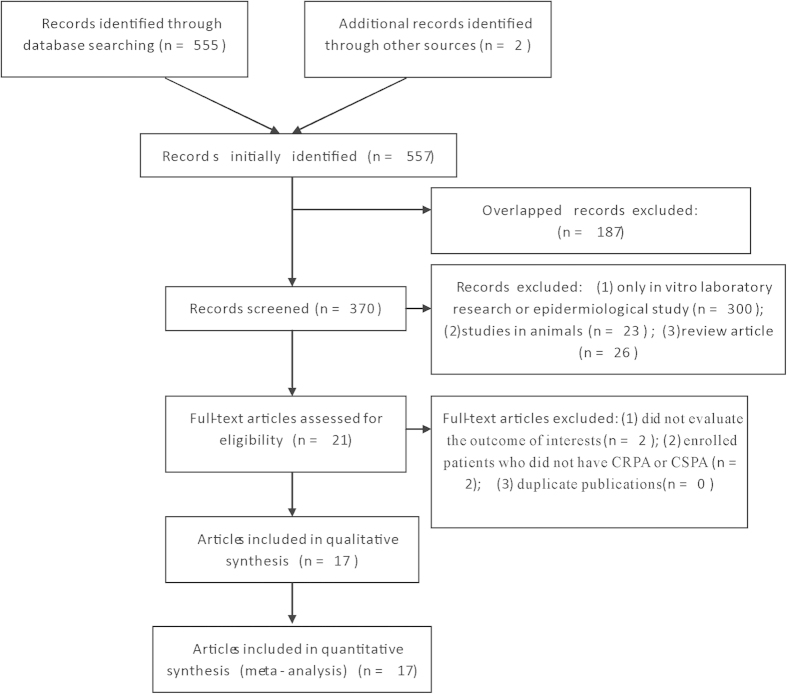
Flow diagram of included studies.

Initially, 557 relevant articles were identified. After excluding those overlapped records between the databases, the remaining 370 abstracts were retrieved for further evaluation. Subsequently, 21 articles with full texts that met the inclusion criteria were assessed. Then four articles were excluded for being not relevant to the influence of carbapenem resistance on mortality of patients with *P. aeruginosa* infection. Hence, a total of 17 studies were included in this meta-analysis (Fig. 1).

### Study characteristics

The basic characteristics of each included study are listed in Table 1^8^,^13^,^14^,^16^,^17^,^18^,^19^,^20^,^21^,^22^,^23^,^24^,^25^,^26^,^27^,^28^,^29^. These 17 studies were published from 1996 to 2014, including 6660 patients carrying *P. aeruginosa*, from hospitals distributed in Asia (three in Korea, one in India, one in China), Europe (one in Slovakia, two in Spain), North America (Seven in USA), and South America (two in Brazil). Carbapenem resistance rates ranged from 8.7% to 50.4%, showing the overall trend to be on the rise over the time and obviously different distribution among various regions. Among those researches, three were designed as matched case-control studies, while 14 belonged to cohort studies. 10 studies focused on bacteraemia as the source of infections, while the other seven studies aimed at any infection or colonization with *P. aeruginosa*.

The results of all included studies are shown in Supplementary Table S1. Totally, 16 studies reported crude odds ratios (OR) or risk ratio (RR) concerning the influence of carbapenem resistance on mortality of *P. aeruginosa* infection, while only four studies provided the adjusted OR or RR. As inappropriate antimicrobial treatment and severity of underlying diseases were generally regarded as the leading confounding factors affecting both the risk of antibiotic resistance and mortality, we also attempted to assess these variables. When comparing patients infected with CRPA and carbapenems susceptible *P. aeruginosa* (CSPA), three studies didn’t detect any difference in severity of underlying diseases, while two studies reported significant differences in inappropriate antimicrobial treatment. Although six studies didn’t find any increased risk for mortality regarding inappropriate antimicrobial treatment in *P. aeruginosa* infection, another study showed higher risk [OR = 5.54; 95% confidence interval (95%CI) = 2.15, 14.56] after adjusting the potential influencing factors in multivariate analysis. Additionally, seven studies reported elevated risk for more severe underlying diseases in *P. aeruginosa* infection, despite that statistical significance was not obvious in two studies.

### Methodological quality

The methodological quality scores of all included studies range from 5 to 9, with 88.24% of the studies (15 of 17) identified to be high quality (Supplementary Table S2 and S3). Two studies scored only five mainly because they didn’t establish a consistent follow-up time for mortality during observation period. What’s more, they didn’t adjust for potential confounders in analyses.

### Statistical results

**Figure 2:**
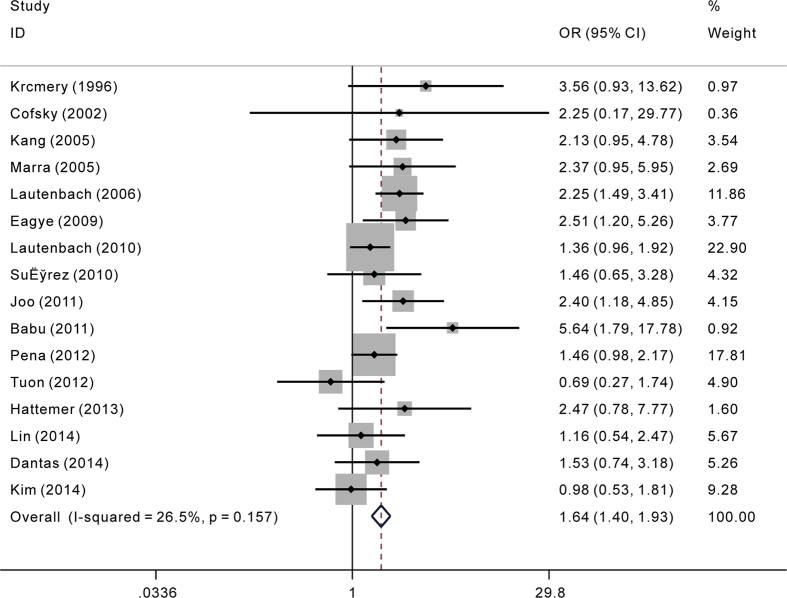
Crude odds ratio (OR) for the association between carbapenem resistance and mortality of patients with *Pseudomonas aeruginosa* infection.

**Figure 3:**
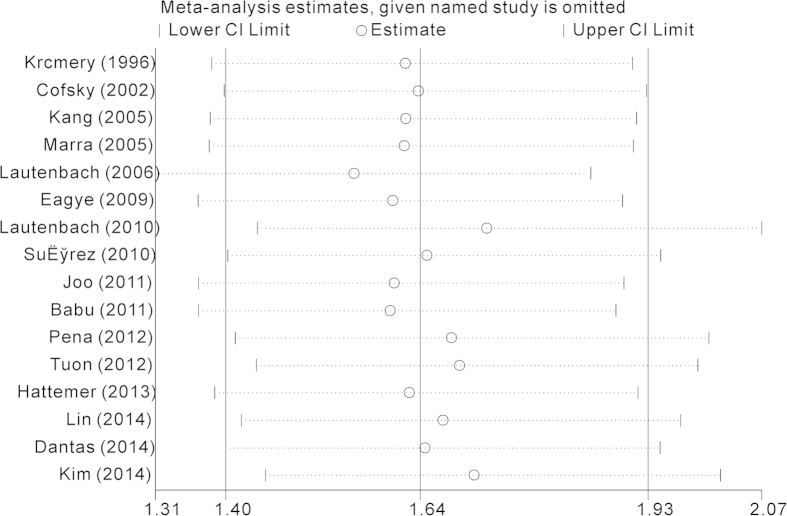
Sensitivity analysis of Crude odds ratio (OR) for the association between carbapenem resistance and mortality of patients with *Pseudomonas aeruginosa* infection.

**Figure 4:**
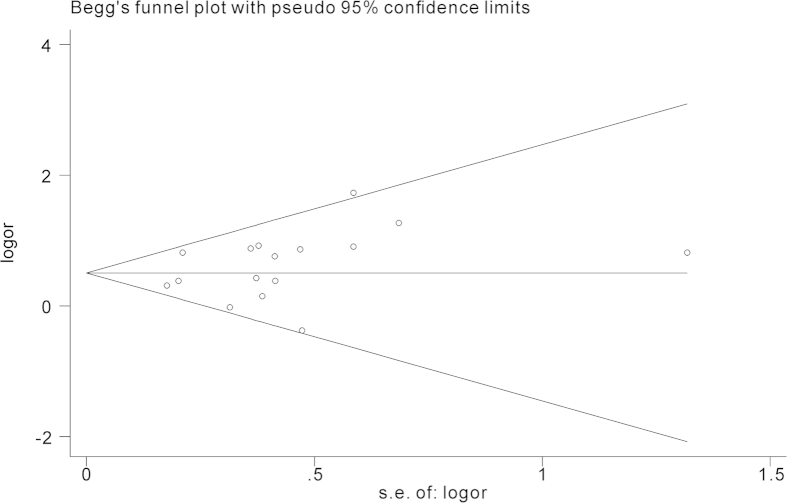
The funnel plot for selected studies on Crude odds ratio (OR) for the association between carbapenem resistance and mortality of patients with *Pseudomonas aeruginosa* infection.

The overall results of the relationship between carbapenem resistance and risk for crude mortality in *P. aeruginosa* infections are presented in Fig. 2. 16 studies involving 5788 patients reported effect estimates in the univariate analysis. No statistical heterogeneity was observed among studies (*p* = 0.157, *I*^*2*^ = 26.5%). The pooling analysis indicated that patients with CRPA had significantly higher mortality than patients with CSPA (crude OR = 1.64; 95%CI = 1.40, 1.93). We further performed subgroup analyses by study design, region, sites of infection, and type of carbapenem respectively (Table 2). The elevated risk of mortality in patients with CRPA infection was not lessened when stratified by the above potential confounding factors, except that the estimate effect conducted in South America proved to be insignificant. During sensitivity analysis, after excluding each study one by one, we got almost the same results (Fig. 3). Begg’s (z = 0.95, p = 0.34) and Egger’s test (t = 1.23, p = 0.24) showed no evidence of publication bias concerning crude ORs (Fig. 4).

**Figure 5:**
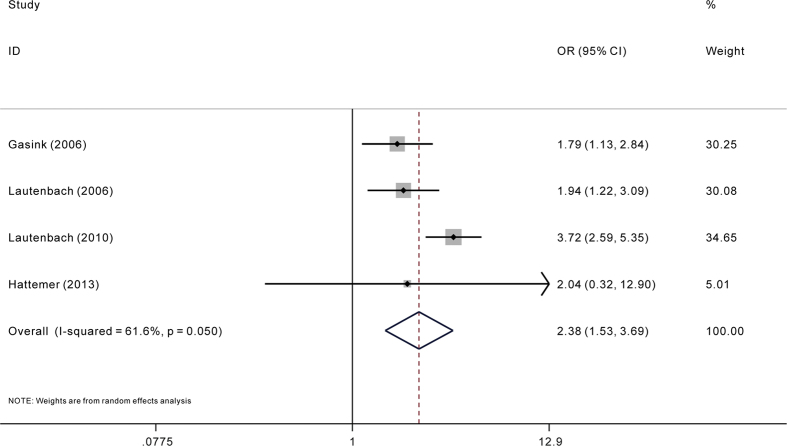
Adjusted odds ratio (OR) for the association between carbapenem resistance and mortality of patients with *Pseudomonas aeruginosa* infection.

**Figure 6:**
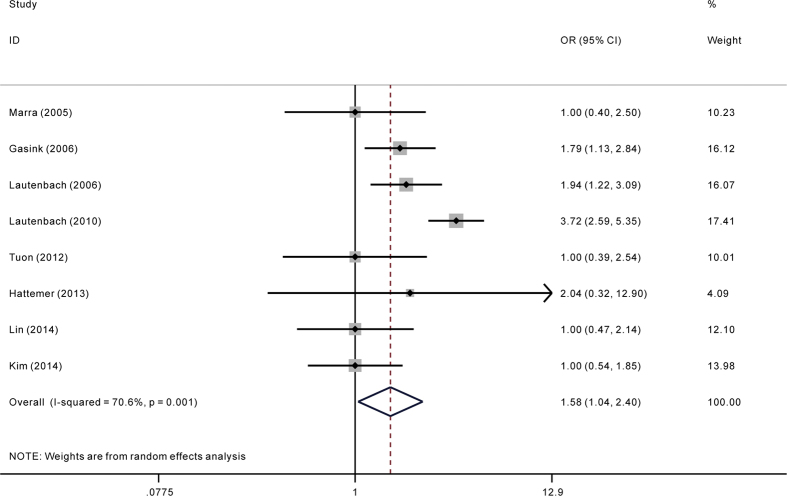
Sensitivity analysis of adjusted odds ratio (OR) for the association between carbapenem resistance and mortality of patients with *Pseudomonas aeruginosa* infecti*on.*

Among all included studies, only five reported adjusted ORs. The pooling analysis of adjusted ORs indicated significant association between carbapenem resistance and mortality risk in *P. aeruginosa* infection (adjusted OR = 2.38; 95%CI = 1.53, 3.69) (Fig. 5). When including studies with assumed adjusted OR in the sensitivity analysis, the trend of increased risk still didn’t changed (adjusted OR = 1.58; 95%CI = 1.04, 2.40) (Fig. 6). For less than ten studies were included in the pooling analysis of adjusted ORs, assessing publication bias were then not carried out in case of inadequacy of power of test.

## Discussion

Treatment of infectious diseases caused by the opportunistic pathogen *P. aeruginosa* is becoming more challenging with each passing year, especially with the advent of carbapenem resistance. CRPA infection was already proved to be independently associated with increased hospital costs and length of hospital stay, even after controlling for important predictors of these outcomes^22^. However, it is still in debate regarding the influence of carbapenem resistance on mortality of *P. aeruginosa* infection. It is considered that many studies had small numbers of patients with *P. aeruginosa* infection, thereby decreasing the statistical power. In the present meta-analysis, patients with CRPA exhibited significantly higher risk of mortality than those with CSPA, when pooling results under univariate analysis or multivariate analysis. Our findings were similar to a recently conducted meta-analysis which aimed to evaluate the influence of carbapenem resistance on mortality in patients infected with another pathogen, *Acinetobacter baumannii* and achieved almost the same tendency of increased mortality in patients with carbapenem-resistant pathogen^30^.

The main reasons for association between carbapenem resistance and elevated mortality are speculated to as follows. Firstly, it is often recognized that infections with CRPA may lead to raised probability of receiving initially inappropriate antimicrobial therapy. The latter were demonstrated to be associated with higher risk for mortality^31^. Secondly, a number of studies reported elevated mortality risk for patients with *P. aeruginosa* infection under more severe underlying diseases. Thus, it is suspected that there may be interaction between carbapenems resistance and the severity of co morbid diseases^31^. However, as is shown in our research, when comparing patients infected with CRPA and CSPA, the majority of included studies didn’t detect any difference in severity of underlying diseases. Beyond that increased virulence in resistant pathogens was also proposed to explain their adverse impact on clinical outcomes^31^. Yet, Bjorkman *et al.* reported that CRPA group had a slower death than the CSPA group in the first 48 h after the bacteraemia, which might suggest a lower virulence of CRPA strains^32^. What’ more, *in vitro* evidence also showed that resistance genes or mutations can alter the fitness of microorganisms, reducing their capacity to generate deleterious host inflammatory responses, which further make it hard to the justify the hypothesis of increased virulence in resistant pathogens^33^. On the whole, it is now still difficult to clearly elucidate the specific reason regarding the elevated mortality in CRPA infection.

We obtained consistent results of increased mortality among CRPA after further stratified analyses by study design, sites of infection, and type of carbapenems. However, in the subgroup of patients from South America, no statistically difference was demonstrated in our research. Regarding this point, it is estimated that differences in virulence of *P. aeruginosa*, antibiotics prescription, medical treatment strategy and environmental factors in different regions may contribute to this negative result^26^.

Carbapenem resistance in *P. aeruginosa* is reported to result from the complex interaction of several mechanisms including the production of carbapenemase, the over-expression of efflux systems, and the loss of outer membrane porins^34^. Among these resistance mechanisms, production of carbapenemase is most important because it occupies a large majority and is associated with higher mortality rate^35^. Along with the increasingly clearer understanding of drug-resistant mechanisms, investigating the clinical outcome of CRPA infection classified by different resistance mechanisms may further promote future study.

The strength of our meta-analysis include exhaustively search strategy as well as large sample size, which may add more statistical power to detect differences and allow for subgroup analyses. However, some limitations should also be addressed. Firstly, some detailed information, such as the inappropriate antimicrobial treatment, the severity of underlying diseases between CRPA and CSPA, and resistance mechanisms in clinical isolates of *P. aeruginosa* was not all available, which limited our further assessment by performing stratified analysis. Secondly, some included studies identified subjects based on the database of clinical culture results, thus not all elements were available to apply CDC criteria fully to determine which of these culture results represented true infection or colonization^36^. Under this situation, blood cultures that yield *P. aeruginosa* would be more reliable to determine true pathogens or colonizers. However, in our subsequent analyses by sources of infection, no substantive differences were discovered. Thirdly, not all included studies in our meta-analysis reported definitions of definite cut off value used to judge the susceptibility of *P. aeruginosa* to imipenem and/or meropenem. Accordingly, there may exist potential heterogeneity. Fourthly, none of our included studies are randomised controlled trial, thus some confounded factors cannot be adjusted, which is a compromise. Lastly, we only included studies published in English, which may lead to the miss of some relevant reports published in other languages.

In conclusion, the pooling analysis indicated that patients infected with CRPA had significantly higher mortality than those infected with CSPA, whether under unvariate or multivariate analysis. However, the impact of CRPA on the mortality in an index particular patient was more or less subjective and should be assessed in the background of other risk factors prevailing in a patient.

## Methods

### Search strategy

We searched the following databases from their inception until December 25, 2014: PUBMED, Web of science, EMBASE, Google Scholar, the Cochrane Library. The search strategies were based on the following terms: ‘aeruginosa’ in combination with ‘mortality’ or ‘death’, and in combination with ‘carbapenem’ or ‘imipenem’ or ‘meropenem’. Furthermore, the reference lists of reports identified by this search strategy were also searched for relevant articles.

### Inclusion and exclusion criteria

Studies were considered eligible for inclusion if they were cohort or case-control studies assessing the impact of carbapenem resistance on mortality of patients with *P. aeruginosa* infection. Carbapenem resistance in this study was defined as resistance to imipenem and/or meropenem (with no limitation on definition of definite cut off value), irrespective of susceptibility to other antibiotics. The other *P. aeruginosa* isolates were considered as CSPA. In-hospital all-cause mortality was defined as the primary clinical outcome in our analysis. Appropriate antimicrobial therapy was defined as at least one of the antibiotics given was later proved to be active against the *P. aeruginosa* isolate *in vitro*.

Researches focusing on animals, referring only to therapeutic effect of carbapenems on *P. aeruginosa* infections, concerning only the *in vitro* susceptibility of carbapenem in *P. aeruginosa* were excluded. In addition, duplicated publications were not included in our statistical analysis. When the same study was included by more than one article, we would select the study with higher sample size. We included published articles only written in English.

### Data abstraction and quality assessment

Two independent investigators (Qianqian Liu and Xiaoqing Li) assumed the task of abstraction of data and discrepancies were solved by discussion and referring to the relevant literatures. The following variables were extracted from original publications: title, first author and year of publication, country of origin, region of study population, study design, sample size, baseline characteristics of the study population (age, sex, source of infection, severity of underlying disease), inappropriate antimicrobial treatment rates, crude mortality rates among patients with CRPA and CSPA, adjusted OR or RR and 95%CI of carbapenems resistance on mortality, variables adjusted in multivariate regression model. If relevant data were not displayed, the investigators would attempt to contact the corresponding author to obtain the missing data.

We assessed the methodological quality of cohort or case-control studies included in the meta-analysis according to the Newcastle-Ottawa scale (NOS) score, ranging from 0 to 9 scores^37^. Studies with NOS score less than three were classified as poor quality and were excluded from this meta-analysis. While studies with NOS score greater than or equal to seven were identified to be of high quality.

### Statistical analysis

Between-study heterogeneity was assessed by the *χ2* based Q statistics and *I*^*2*^ test. Fixed effect model (Mantel–Haenszel’s method) was adopted if the result of the heterogeneity test was *p* > 0.10, otherwise the random effect model (DerSimonian and Laird’s method) was used^38^,^39^. We transformed RR into OR when necessary using equation provided by Zhang *et al.*^40^. OR estimates and 95% CIs on a natural logarithmic scale were used to assess the strength of association between carbapenem resistance and the mortality in patients with *P. aeruginosa* infection in both crude and adjusted analysis. Stratified analyses were performed by region, type of carbapenem, source of infection, and study design. Sensitivity analyses of crude OR were performed to assess the stability of the results by excluding each study one by one. In addition, several studies in our research detected no significant statistical differences in mortality between patients with CRPA and CSPA infections during univariate analysis, thus they didn’t conducted the risk estimate in subsequent multivariate analysis. As excluding those studies in the pooling analysis of adjusted OR may introduce potential bias, we included them in the sensitivity analysis, assuming adjusted OR to be 1 and calculated the 95% CIs from the univariate analysis^30^. Begg’s and Egger’s tests were used to test the possible publication bias when more than ten studies were pooled^41^. All statistical analyses were done with STATA version 12.0 (StataCorp, College Station, Texas), using metan programme and two-sided p values.

## Additional Information

**How to cite this article**: Liu, Q. *et al.* Influence of carbapenem resistance on mortality of patients with *Pseudomonas aeruginosa* infection: a meta-analysis. *Sci. Rep.* **5**, 11715; doi: 10.1038/srep11715 (2015).

## Supplementary Material

Supplementary Information

## Figures and Tables

**Table 1:** Characteristics of studies included in meta-analysis.

**Author (year)**	**Country**	**Type of study**	**Sample size, n: CRPA vs. CSPA**	**Sites of infection**	**Definition of resistance**	**Type of carbapenem**	**Carbapenem Resistance**	**NOS score**
Krcmery (1996)^15^	Slovakia	Retrospective cohort	10 vs. 91	Bacteremia	NA	Imipenem	9.9%	5
Cofsky (2002)^16^	USA	Retrospective case-control	10 vs. 10	Any infection	NA	Carbapenem	NA	9
Kang (2005)^17^	Korea	Retrospective cohort	28 vs. 162	Bacteremia	NCCLS	Imipenem	14.7%	7
Marra (2005)^18^	USA	Retrospective cohort	16 vs. 72	Bacteremia	NA	Imipenem	18.2%	7
Gasink (2006)^8^	USA	Retrospective cohort	872	Any infection or colonization	NCCLS	Imipenem	NA	7
Lautenbach (2006)^19^	USA	Retrospective cohort	135 vs. 719	Any infection or colonization	CLSI	Imipenem	15.8%	7
Eagye (2009)^20^	USA	Retrospective case-control	58 vs. 125	Any infection or colonization	NA	Meropenem	NA	9
Lautenbach (2010)^21^	USA	Retrospective cohort	253 vs. 2289	Any infection or colonization	CLSI	Imipenem	10.0%	9
Suárez (2010)^22^	Spain	Retrospective cohort	33 vs. 88	Bacteremia	CLSI (2005)	Carbapenem	27.3%	9
Joo (2011)^13^	Korea	Retrospective cohort	46 vs. 156	Bacteremia	NA	Imipenem	22.8%	9
Babu (2011)^23^	India	Prospective cohort	24 vs. 86	Any infection	CLSI (2007)	Imipenem	21.8%	5
Pena (2012)^24^	Spain	Prospective cohort	145 vs. 487	Bacteremia	CLSI	Carbapenem	22.9%	9
Tuon (2012)^25^	Brazil	Retrospective cohort	29 vs.48	Bacteremia	CLSI (2010)	Carbapenem	37.7%	9
Hattemer (2013)^14^	USA	Retrospective cohort	13 vs. 137	Bacteremia	CLSI (2012)	Carbapenem	8.7%	9
Lin (2014)^26^	China	Retrospective case-control	82 vs. 82	Any infection or colonization	CLSI (2011)	Carbapenem	NA	7
Dantas (2014)^27^	Brazil	Retrospective cohort	55 vs. 65	Bacteremia	CLSI	Carbapenem	45.8%	7
Kim (2014)^28^	Korea	Retrospective cohort	118 vs. 116	Bacteremia	CLSI (2008)	Carbapenem	50.4%	9

Abbreviations: CRPA, carbapenem-resistant *Pseudomonas aeruginosa*; CSPA, carbapenem-susceptible *Pseudomonas aeruginosa*; NOS, Newcastle-Ottawa Scale; NA, not available; NCCLS, National Committee for Clinical Laboratory Standards; CLSI, Clinical and Laboratory Standard Institute.

**Table 2:** Subgroup analyses of studies included in meta-analysis.

**Factors**	**Subgroups**	**Number of included studies**	**Crude OR(95%CI)**	**Statistical model**	***I***^***2***^	***P***_**heterogeneity**_
Study design	Case-control study	3	1.72(1.03–2.89)	Fixed	4.1%	0.352
	Cohort study	13	1.63(1.38–1.93)	Fixed	34.3%	0.108
Sources of infection	Bacteraemia	10	1.54(1.23–1.93)	Fixed	11.6%	0.336
	Any infection or colonization	6	1.92(1.31-2.81)	Random	47.7%	0.089
Region	Europe	3	1.55(1.10–2.18)	Fixed	0.0%	0.454
	Asia	5	1.82(1.06–3.11)	Random	57.8%	0.05
	North America	6	1.82(1.44–2.29)	Fixed	1.8%	0.405
	South America	2	1.12(0.64–1.99)	Fixed	43.5%	0.183
Type of carbapenem	Carbapenem	8	1.30(1.02–1.67)	Fixed	0.0%	0.698
	Meropenem	1	2.51(1.20–5.26)	Fixed	NA	NA
	Imipenem	7	1.92(1.54–2.39)	Fixed	35.0%	0.161

Abbreviations: NA, not available.
