# Supplementary material for: Influence of carbapenem resistance on mortality of patients with Pseudomonas aeruginosa infection: a meta-analysis
Source: Sci Rep. 2015 Jun 25;5:11715. doi: 10.1038/srep11715 (PMC4479982; doi:10.1038/srep11715)
Supplement: Supplementary Information [file srep11715-s1.doc]

**Influence of carbapenem resistance on mortality of patients with *Pseudomonas aeruginosa* infection: a meta-analysis**

**Qianqian Liu^1,+^, Xiaoqing Li^2,+^, Wenzhang Li^3^, Xinmiao Du^4^, Jian-Qing He^4^, Chuanmin Tao^1,*^, Yulin. Feng^4,*^**

^1^Department of Laboratory Medicine, West China Hospital of Sichuan University, Chengdu, Sichuan, 610041, China.

^2^Department of yanglao and old age collaborative innovation, Chengdu Medical College, Chengdu, Sichuan, 610500, China.

^3^Department of Cardiology, First Affiliated Hospital of Chengdu Medical College, Chengdu, Sichuan, 610500, China.

^4^Department of Respiratory Medicine, West China Hospital of Sichuan University, Chengdu, Sichuan, 610041, China.

^*^corresponding authors: Chuanmin Tao (chuan112500@126.com) and Yulin. Feng ([hx399@163.com](mailto:hx399@163.com))

^+^these authors contributed equally to this work

**Supplementary Table S1.** Results of studies included in meta-analysis

| Author (year) | Crude mortality rate: CRPA vs. CSPA | Adjusted OR or RR | Variables adjusted in the multivariable model | Inappropriate antimicrobial treatment: CRPA vs. CSPA | Inappropriate antimicrobial treatment and risk of mortality | Severity of underlying diseases: CRPA vs. CSPA | Severity of underlying diseases and risk of mortality |
| --- | --- | --- | --- | --- | --- | --- | --- |
| Krcmery (1996) | 6/10 vs. 27/91 | NA | Not adjusted | NA | NA | NA | NA |
| Cofsky (2002) | 2/10 vs. 1/10 | NA | Not adjusted | NA | NA | NA | NA |
| Kang (2005) | 15/28 vs. 57/162 | NA | Not adjusted | NA | NA | NA | NA |
| Marra (2005) | 16/26 vs. 19/72 | NA Not significant | Not adjusted | NA | OR=1.13(0.51-2.52) | NA | APACHE II score adjusted OR=9.7(1.9–47.9) |
| Gasink (2006) | NA | adjusted OR=1.79 (1.13, 2.84) | Adjusted by Fluoroquinolone Resistance and Duration of hospitalization | NA | NA | NA | NA |
| Lautenbach (2006) | 42/ 135 vs. 120/719 | adjusted OR=1.94(1.22-3.10) | Adjusted by in ICU when culture yielded an imipenem-resistant isolate, bloodstream involvement of infection with imipenem-resistant isolate, Charlson Comordity Index, Hospitalization duration | NA | NA | NA | Charlson Index adjusted OR=1.06(1.01-1.12) |
| Eagye (2009) | 18/58 vs. 19/125 | NA | adjusted for time at risk | NA | NA | NA | NA |
| Lautenbach (2010) | 44/253 vs. 307/2289 | adjusted OR=3.72 (2.59–5.35) | Adjusted for patient location in an ICU, transfer from another health-care facility, and duration of hospitalization | NA | NA | NA | NA |
| Suárez (2010) | 15/33 vs. 32/88 | NA | Adjusted for age, sex and Charlson index | p=0.004 | OR=0.85(0.37-1.96) | Charlson index (p=0.71) | Charlson index RR=1.5(0.9–2.2) |
| Joo (2011) | 18/46 vs. 33/156 | NA | Not adjusted | NA | OR=0.88(0.46-1.69) | NA | Charlson index adjusted OR=1.31(1.04-1.65) |
| Babu (2011) | 8/24 vs. 7/86 | NA | Not adjusted | NA | NA | NA | NA |
| Pena (2012) | 51/145 vs. 132/487 | NA | Adjusted by the source of bacteraemia, the Pitt score, and a delay of less than 24 h to receive adequate antibiotic therapy | p<0.001 | OR=1.25(0.88-1.78) | Charlson index (p=0.23) | Charlson index was significant risk factor for mortality |
| Tuon (2012) | 13/29 vs. 26/48 | NA Not significant | Not considered for multivariate analysis | NA | NA | NA | NA |
| Hattemer (2013) | 7/13 vs. 44/137 | adjusted RR=1.53(0.68-3.42) | Adjusted by SOFA score at the time of bacteraemia, TTS phenotype, sites of infection acquired | NA | OR=1.20(0.49-2.97) | Charlson index (p=0.65) | Charlson index was not significant risk factor for mortality |
| Lin (2014) | 18/82 vs. 16/82 | NA Not significant | Not considered for multivariate analysis | NA | NA | NA | NA |
| Dantas (2014) | 26/55 vs. 24/65 | NA | Not adjusted | p=0.10 | adjusted OR=5.54 (2.15-14.56) | NA | ASIS score ≥ 4 OR=1.54 (0.69-3.45) |
| Kim (2014) | 26/118 vs. 26/116 | NA Not significant | Not considered for multivariate analysis | NA | NA | NA | APACHE II score adjusted OR=4.65(1.95-11.04) |

Abbreviations: CRPA, carbapenem-resistant *Pseudomonas aeruginosa*; CSPA, carbapenem-susceptible *Pseudomonas aeruginosa*; OR, odds ratio; RR, relative risk; NA, not available; ICU, intensive care unit; SOFA, Sequential Organ Failure Assessment; TTS, type III secretory; ASIS, Average Severity of Illness Score.

**Supplementary Table S2.** Newcastle-Ottawa scale (NOS) score for case control studies

| Author (year) | Is the case definition adequate? | Representativeness of the cases | Selection of Controls | Definition of Controls | Comparability of cases and controls on the basis of the design or analysis | Ascertainment of exposure | Same method of ascertainment for cases and controls | Non-Response rate |
| --- | --- | --- | --- | --- | --- | --- | --- | --- |
| Cofsky (2002) | **🟑** | **🟑** | **🟑** | **🟑** | **🟑🟑** | **🟑** | **🟑** | **🟑** |
| Eagye (2009) | **🟑** | **🟑** | **🟑** | **🟑** | **🟑🟑** | **🟑** | **🟑** | **🟑** |
| Lin (2014) | **🟑** | **🟑** | **🟑** | **🟑** | - | **🟑** | **🟑** | **🟑** |

**Supplementary Table S3.** Newcastle-Ottawa scale (NOS) score for cohort studies

| Author (year) | Representativeness of the exposed cohort | Selection of the non exposed cohort | Ascertainment of exposure | Demonstration that outcome of interest was not present at start of study | Comparability of cohorts on the basis of the design or analysis | Assessment of outcome | Was follow-up long enough for outcomes to occur | Adequacy of follow up of cohorts |
| --- | --- | --- | --- | --- | --- | --- | --- | --- |
| Krcmery (1996) | **🟑** | **🟑** | **🟑** | **🟑** | - | **🟑** | - | - |
| Kang (2005) | **🟑** | **🟑** | **🟑** | **🟑** | - | **🟑** | **🟑** | **🟑** |
| Marra (2005) | **🟑** | **🟑** | **🟑** | **🟑** | **🟑🟑** | **🟑** | - | - |
| Gasink (2006) | **🟑** | **🟑** | **🟑** | **🟑** | **🟑🟑** | **🟑** | - | - |
| Lautenbach (2006) | **🟑** | **🟑** | **🟑** | **🟑** | **🟑🟑** | **🟑** | - | - |
| Lautenbach (2010) | **🟑** | **🟑** | **🟑** | **🟑** | **🟑🟑** | **🟑** | **🟑** | **🟑** |
| Suárez (2010) | **🟑** | **🟑** | **🟑** | **🟑** | **🟑🟑** | **🟑** | **🟑** | **🟑** |
| Joo (2011) | **🟑** | **🟑** | **🟑** | **🟑** | **🟑🟑** | **🟑** | **🟑** | **🟑** |
| Babu (2011) | **🟑** | **🟑** | **🟑** | **🟑** | - | **🟑** | - | - |
| Pena (2012) | **🟑** | **🟑** | **🟑** | **🟑** | **🟑🟑** | **🟑** | **🟑** | **🟑** |
| Tuon (2012) | **🟑** | **🟑** | **🟑** | **🟑** | **🟑🟑** | **🟑** | **🟑** | **🟑** |
| Hattemer (2013) | **🟑** | **🟑** | **🟑** | **🟑** | **🟑🟑** | **🟑** | **🟑** | **🟑** |
| Dantas (2014) | **🟑** | **🟑** | **🟑** | **🟑** | - | **🟑** | **🟑** | **🟑** |
| Kim (2014) | **🟑** | **🟑** | **🟑** | **🟑** | **🟑🟑** | **🟑** | **🟑** | **🟑** |
